# Application of a Plug-and-Play Immunogenicity Assay in Cynomolgus Monkey Serum for ADCs at Early Stages of Drug Development

**DOI:** 10.1155/2016/2618575

**Published:** 2016-03-22

**Authors:** Montserrat Carrasco-Triguero, Helen Davis, Yuda Zhu, Daniel Coleman, Denise Nazzal, Paul Vu, Surinder Kaur

**Affiliations:** ^1^BioAnalytical Sciences, Genentech, Inc., 1 DNA Way, South San Francisco, CA 94080, USA; ^2^Biostatistics (MDBB), Genentech, Inc., 1 DNA Way, South San Francisco, CA 94080, USA

## Abstract

Immunogenicity assessment during early stages of nonclinical biotherapeutic development is not always warranted. It is rarely predictive for clinical studies and evidence for the presence of anti-drug antibodies (ADAs) may be inferred from the pharmacokinetic (PK) profile. However, collecting and banking samples during the course of the study are prudent for confirmation and a deeper understanding of the impact on PK and safety. Biotherapeutic-specific ADA assays commonly developed can require considerable time and resources. In addition, the ADA assay may not be ready when needed if the study of PK and safety data triggers assay development. During early stages of drug development for antibody-drug conjugates (ADCs), there is the added complication of the potential inclusion of several molecular variants in a study, differing in the linker and/or drug components. To simplify analysis of ADAs at this stage, we developed plug-and-play generic approaches for both the assay format and the data analysis steps. Firstly, the assay format uses generic reagents to detect ADAs. Secondly, we propose a cut point methodology based on animal specific baseline variability instead of a population data approach. This assay showed good sensitivity, drug tolerance, and reproducibility across a variety of antibody-derived biotherapeutics without the need for optimization across molecules.

## 1. Introduction

All biotherapeutics, including antibody-drug conjugates (ADCs), have the potential to elicit an immune response in humans that could impact their efficacy, pharmacokinetics, and safety. Hence, the assessment of immunogenicity is a key component during clinical development as well as a regulatory requirement [1–4]. ADCs for oncology indications are composed of a cytotoxic drug linked to a monoclonal antibody (mAb) that recognizes a tumor-associated antigen. Although ADCs contain structural motifs that may increase their immunogenicity, they can nevertheless follow the immunogenicity and assay strategies used for other biotherapeutics with some modifications [5–7].

In a nonclinical setting, it is expected that human protein therapeutics elicit an immune response in animal species. Differences in protein sequences between humans and nonclinical species together with other product related factors contribute to this immune response [8]. Immunogenicity in animals is generally not predictive of immunogenicity in humans and evaluations in nonclinical studies are not always warranted [9]. However, collecting and banking samples during the course of the study are recommended to ensure samples are available if future analysis is needed to explain the pharmacokinetics (PK), exposure, and/or safety data from the study.

Immunogenicity in animal species is normally evaluated by detecting anti-drug antibodies (ADAs) in circulation. Immunoassay-based technologies are widely used for this purpose [8] with technologies such as mass spectrometry emerging in this arena [10]. Detection of ADAs requires the use of the biotherapeutic as a reagent, which for some immunoassay formats involves conjugation to specific labels (e.g., biotin, ruthenium, digoxigenin, and Alexa Fluor® dyes). Assay development, qualification, and validation require ADA surrogate controls to characterize the performance of the assay. ADA controls for nonclinical assays can be either biotherapeutic-specific or generic, anti-human IgG polyclonal, or monoclonal antibodies. The threshold to determine positivity for biotherapeutic-specific assays is usually established based on the population variability by the analysis of samples from nontreated naive individuals [11, 12].

Our nonclinical immunogenicity strategy for ADC lead candidates selected for preclinical development includes developing ADC-specific ADA assays to support PK and toxicity studies in cynomolgus monkeys [5]. However, there are some caveats with this approach when a program is at the discovery stage. Often a variety of candidate molecules may be evaluated in the same study. In the case of ADCs, these studies may include candidates with different linkers and/or small molecule drugs. In addition, a small number of animals may be used to evaluate each candidate. At this early stage of drug development, the development of molecule specific ADA assays for each candidate could be laborious and resource intensive. Moreover, if the samples are banked and the analysis is triggered by the need to understand PK and/or safety data, developing an assay at that time could impact the ability to make key decisions for the program in a timely manner.

For ADCs in research, our immunogenicity strategy for most PK and safety studies in cynomolgus monkeys is to collect and bank the samples. Having a nonclinical immunogenicity assay applicable across all ADCs would be beneficial to enabling streamlined ADA evaluation across all candidate molecules. The key requirements for such an assay would be readily available capture and detection reagents either in-house or from vendors, a universal assay positive control, ability to detect ADAs to all domains of an ADC, appropriate sensitivity, drug tolerance, and no need for assay optimization with each ADC molecule. In addition to the assay format, cut points or thresholds to determine ADA positivity should be the same for all molecules. Generic or universal assay formats to detect ADAs against mAb biotherapeutics in nonclinical species have been described by others [13, 14]. These types of assays could be also applied to ADCs.

In this paper, we present a generic ADA assay format in cynomolgus monkey serum modified from the one described by Stubenrauch et al. [13] to determine ADAs to ADCs. In addition, we describe a cut point methodology based on animal specific baseline variability instead of the commonly used population data approach. This assay relies on the detection of complexes formed between ADAs and the biotherapeutic. The specific biotherapeutic was added to the samples to ensure that ADAs not complexed to the biotherapeutic in the sample were captured. Capture and detection reagents as well as assay controls either were readily available in our laboratory, were purchased from vendors, or were easy to produce. In addition to generic reagents, the positivity of a sample was determined by a simple cut point methodology specific to each animal instead of a biotherapeutic-specific population approach. As there is no need for assay optimization across molecules, sample testing from programs in preclinical research can be quick. This is a desirable attribute when analysis of banked samples is only triggered by the need to understand PK and/or safety data. This assay was reproducible and showed appropriate sensitivity and drug tolerance. Samples from PK studies in cynomolgus monkeys with two ADCs were analyzed in the plug-and-play assay. The data were generally similar to those previously obtained using our default ADC-specific ELISA. The plug-and-play assay was superior in detecting more incidences of positive responses and earlier on.

## 2. Materials and Methods

### 2.1. Buffers, Reagents, Serum Samples, and Test Molecules

The capture reagent for the plug-and-play assay was a murine IgG1 monoclonal antibody R10Z8E9 [13, 15, 16] generated at Genentech, a member of the Roche group (South San Francisco, CA, USA), and is also available from various commercial sources. This antibody is directed against a conformation epitope on the CH2 domain of all four subclasses of human Fc gamma [17]. The detection reagent was a horseradish peroxidase- (HRP-) conjugated goat anti-monkey IgG polyclonal antibody against heavy and light chain with minimal reactivity to human IgG (Bethyl Laboratories Inc., Montgomery, TX, USA). The anticytotoxic drug ADA proof of concept assay used a sheep anti-human IgG (Binding Site, San Diego, CA, USA) as capture reagent and an HRP-conjugated goat anti-mouse IgG antibody (KPL Inc., Gaithersburg MD, USA) for detection.

All the test molecules were generated at Genentech (South San Francisco, CA, USA). These included 4 ADCs, a one-arm antibody produced using knob and hole technology (mAb 1), four recombinant humanized IgG1 mAbs (mAbs 2, 3, 5, and 6), and one recombinant humanized IgG4 mAb (mAb 4). ADCs A, B, C, and D were composed of humanized IgG1 mAbs binding to different targets and sharing the same cleavable peptide linker and cytotoxic drug.

Cynomolgus monkey serum pool and serum from individual animals were purchased from Bioreclamation IVT (Westbury, NY, USA). Baseline samples taken one week apart from 15 cynomolgus monkeys were obtained from a Genentech study conducted at Charles River Laboratories (Reno, NV, USA). Positive controls from three sources were used: human IgG-cyno IgG fusion molecule produced for Genentech at R&D Systems (Minneapolis, MN), purified cynomolgus monkey anti-human IgG1 polyclonal Ab from a cynomolgus monkey hyperimmunized with a Genentech mAb-derived biotherapeutic (Genentech Inc.), and two mouse anticytotoxic drug monoclonal antibodies also produced at Genentech.

Other reagents included biotin (Thermo Fisher Scientific, MA, USA); digoxigenin ([DIG] Invitrogen, NY, USA); bovine serum albumin ([BSA] Equitech-Bio Inc., Kerrville, TX, USA); HRP-conjugated mouse antidigoxin mAb (Jackson ImmunoResearch Labs Inc., PA, USA); tetramethylbenzidine ([TMB] KPL Inc., Gaithersburg, MD, USA).

### 2.2. Cynomolgus Monkey Study Samples

Cynomolgus monkey serum study samples were obtained from Covance Laboratories (Madison, WI, USA). In Study 1, cynomolgus monkeys received ADC A or the corresponding monoclonal antibody A at a single dose of 3 mg/kg, with four monkeys in each arm. Samples for ADA analysis were collected at baseline and on study days 15 and 44 after baseline. In Study 2, cynomolgus monkeys received a single dose of ADC B (0.3 and 1 mg/kg) or the corresponding monoclonal antibody B (1 mg/kg), with three animals in each arm. Samples for ADA were collected at baseline and on study day 43 after baseline.

### 2.3. Biotherapeutic-Specific ADA ELISA

**Figure 1:**
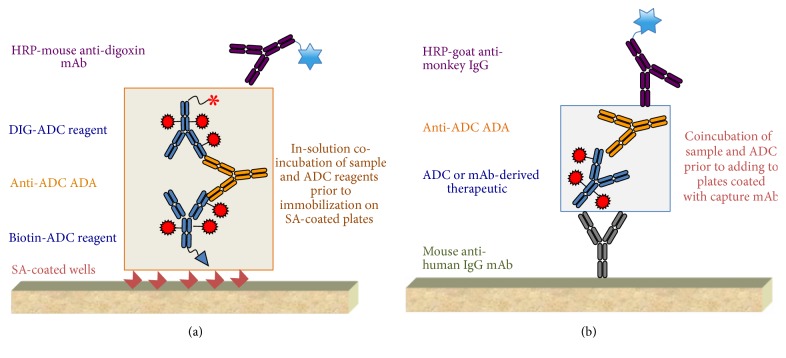
ADA assay formats used for cynomolgus monkeys. (a) Biotherapeutic-specific biotin-DIG ELISA used to screen ADAs in samples from two cynomolgus studies with different ADCs. In this assay, the specific ADC reagents conjugated to biotin or digoxigenin are incubated with the samples. The complexes are immobilized using streptavidin-coated plates and then detected using a mouse antidigoxin mAb conjugated to HRP. (b) Generic ADA assay format developed for ADCs and other mAb-derived biotherapeutics, also used for analysis of samples from two studies in cynomolgus monkey with different ADCs. In this generic assay, the samples are incubated with the specific biotherapeutic and the complexes immobilized onto plates coated with a mouse anti-human IgG mAb. A goat anti-monkey IgG polyclonal antibody conjugated to HRP is used for detection of the immune complexes. ADC = antibody-drug conjugate; ADAs = anti-drug antibodies; DIG = digoxigenin; ELISA = enzyme-linked immunosorbent assay; HRP = horseradish peroxidase; mAb = monoclonal antibody; SA = streptavidin.

Our current platform for nonclinical ADA assays utilizing biotherapeutic-specific assay reagents is an in-solution bridging enzyme-linked immunosorbent assay (ELISA) [5, 18]. This assay was used to evaluate immune responses in the two studies selected for comparing with the plug-and-play assay.  Figure 1(a) shows a representation of the assay. Briefly, a mixture of biotinylated-biotherapeutic and digoxigenin- (DIG-) biotherapeutic conjugates was diluted in assay diluent (phosphate-buffered saline (PBS), 0.5% BSA, 0.05% polysorbate 20, and 0.05% ProClin® 300 at pH 7.4) and added to each well of a 96-well round bottom polypropylene microtiter plate (Costar/Corning). Then an equal volume of the test samples and controls diluted in assay diluent were added to appropriate wells and incubated overnight while shaking at room temperature. A mixture of diluted samples or controls with the biotherapeutic-conjugated reagents was then added to prewashed streptavidin-coated plates and incubated for 2 hours. After a wash (PBS, 0.05% polysorbate 20 at pH 7.4), HRP-mouse antidigoxin mAb was added to each well and incubated for another hour at room temperature. The peroxidase substrate TMB was added to develop color. The enzymatic reaction was stopped with phosphoric acid and the plates were read on a plate reader at 450 nm to detect absorbance and at 630 nm for reference absorbance.

For each ADC, specific ADC-conjugated reagents were prepared. A floating screening cut point based on the population biological variation and using a negative control for normalization was established for each ADC targeting a 5% nontreated positive rate [12]. Samples that screened positive were serially diluted and analyzed in the assay to obtain ADA relative levels by titer. The titer was expressed as the log⁡10 of the sample dilution whose signal was equal to the cut point signal.

### 2.4. Generic ADA Assay in Cynomolgus Monkey Serum

In the generic assay represented in Figure 1(b), 100 *μ*L of mouse IgG1 monoclonal antibody, R10Z8E9 at 2 *μ*g/mL in coating buffer (PBS, pH 7.2), was added to the 96-well microtiter plates and incubated overnight at 4°C. Test samples were diluted to 10-fold in assay diluent (PBS/pH 7.4/0.5% BSA/0.05% polysorbate 20/0.05% ProClin 300/0.25% CHAPS/0.35 M NaCl). The test molecules (ADCs or mAb-derived biotherapeutics) were diluted to 0.5 *μ*g/mL in assay diluent. The diluted samples and the biotherapeutic were mixed in 1 : 1 ratio and incubated for 2 hours with a final 20-fold dilution of the test sample and 0.25 *μ*g/mL in-well concentration of the biotherapeutic. The coated plates were blocked (PBS/pH 7.4/0.5% BSA/0.05% polysorbate 20/0.05% ProClin 300) and washed (PBS/pH 7.4/0.05% polysorbate 20). Then, the sample mixture was added at 100 *μ*L per well in duplicate. The assay controls, human IgG-cyno IgG fusion positive control, and the pooled cynomolgus monkey serum negative control were directly diluted to 20-fold in assay diluent and added to the coated plates. In some experiments (e.g., sensitivity assessment), further 2-fold dilutions of the positive control were performed in the assay diluent supplemented with 5% pooled cynomolgus monkey serum. After 1-hour incubation followed by another wash step, wells were incubated with 100 *μ*L of the HRP-conjugated goat anti-monkey IgG at 25 ng/mL in assay diluent. After washing, the peroxidase substrate, TMB, was added for the color to develop. The enzymatic reaction was stopped with phosphoric acid. The plates were read on a BioTek ELx405 plate reader (BioTek, Vt, USA) at 450 nm to detect absorbance and at 630 nm for reference absorbance.

The relative levels of ADAs for each animal with postbaseline positive signals were estimated from the screening run data by the ratio of the postbaseline signal to the animal baseline signal. With this approach, it is important to ensure that the sample signals are within the spectrophotometer range prior to calculating the ratios.

### 2.5. Determination of Generic Biotherapeutic Concentration Needed for Immune Complex Formation

To establish the level of biotherapeutic added to the samples for immune complex formation prior to analysis in the assay, 4 ADCs and 4 antibody-derived biotherapeutics were evaluated. The biotherapeutics were spiked at concentrations of 0, 1.25, 2.5, 5.0, 10, and 20 *μ*g/mL in pooled cynomolgus monkey serum and were also supplemented with ADAs (purified cynomolgus monkey anti-human IgG antibody) at concentrations of 0, 0.5, 5, and 50 *μ*g/mL. For each ADA concentration, the response curves and the signal-to-background ratios were evaluated across each biotherapeutic level range. The generic concentration of biotherapeutic was established based on the signal-to-background curves obtained for all the biotherapeutics tested in this experiment. As the generic biotherapeutic concentration would be added to each test sample, its effect on background signal was also assessed for each biotherapeutic.

### 2.6. Determination of Individual Cut Points

Background responses were measured in 30 biotherapeutic naive serum samples from 15 cynomolgus monkeys; two baseline samples from each animal were collected one week apart. Four biotherapeutic molecules were used in the generic cynomolgus ADA assay format for this evaluation: ADC C, mAb 1, mAb 2, and mAb 4. A total of 120 biotherapeutic spiked samples were tested in the assay in duplicate at the minimum dilution. The absorbance value for each sample replicate was reported for statistical analysis.

### 2.7. Statistical Methods to Determine Individual Cut Point

Background signal data from the two baseline samples obtained from all 15 cynomolgus monkeys with the 4 biotherapeutics were used to estimate the within monkey baseline variability. For each sample replicate, normalized scores were calculated as the log (sample signal/negative control signal). A one-way Analysis of Variance (ANOVA) was fit with normalized scores as the dependent variable and monkeys as the independent variable. The residual error of the model, denoted as SD, was used to estimate the within monkey variability. Separately, a one-way ANOVA was also fit for each subset of cynomolgus monkeys specific to each of the 4 molecules.

The generic cut point factor (CPF) was defined as

(1) $$\begin{array}{c} CPF=\exp\left(\;Z_{1-\alpha }SD\;\right), \end{array}$$

where *Z* is standard score for the standard normal, *α* is the false positive rate, and SD is the overall estimate of the within monkey variability.

The individual optical density (OD) signal cut point (CP_*i*_) for cynomolgus monkey *i* was determined by

(2) $$\begin{array}{c} {CP}_{i}={\bar{x}}_{i}*CPF, \end{array}$$

where ${\bar{x}}_{i}$ is the mean of monkey *i*'s baseline signal measurements. A monkey was deemed ADA positive if a postbaseline measurement exceeds CP_*i*_.

Individual OD cut points determined this way are only valid when a monkey baseline and postbaseline samples are run on the same assay plate.

### 2.8. Analysis of Study Samples from Cynomolgus Monkey Studies with Two ADCs

The ability of the plug-and-play assay to detect ADAs in study samples was evaluated by analyzing samples from PK studies in cynomolgus monkeys with two ADCs. Immunogenicity data from these two studies with the ADC-specific biotin-DIG ELISA were available. In Study 1, the cynomolgus was dosed with ADC A or the corresponding mAb A. Samples for immunogenicity evaluation were taken at the baseline and at postbaseline days 15 and 44. In Study 2, the cynomolgus monkeys were dosed with ADC B or the corresponding mAb B. Samples for immunogenicity evaluation were taken at baseline and postbaseline day 43.

The samples from the two studies were analyzed in the plug-and-play screening assay and if positive, the relative levels of ADAs were calculated. The results from the plug-and-play assay were compared to those obtained in the biotherapeutic-specific biotin-DIG ELISA in terms of ADA incidence and the relative levels. In the biotin-DIG ELISA, incidence is defined as the sum of enhanced responses and induced responses. In this assay, the cut point determination used a population approach with a 5% false positive rate so it was possible to detect positive signals at baseline. In such a situation, animals with positive signals at baseline could display increased signal at postbaseline time points, which would be considered enhanced by exposure to the biotherapeutic. In the case of animals with positive signals only after treatment, the ADA responses were considered induced by the exposure to the biotherapeutic. However, in the plug-and-play assay, these types of responses are not distinguished as only signals above the individual animal baseline cut point are considered positive.

## 3. Results and Discussion

### 3.1. Generic ADA Screening Assay Format Selection

Several factors were considered in selecting the generic ADA screening assay format in cynomolgus monkey serum. The first consideration was the required data output for the analysis. For our purpose, screening samples to determine if a positive response was produced after exposing the animals to the biotherapeutic and, if positive, a measure of the relative levels of ADAs were the key goals. The potential of the assay format to be used for confirmation of positive responses or further characterization of the ADAs was not a part of the initial goals. The next consideration was the necessity for reagents to be easy to produce and available either in-house or commercially, including capture and detection reagents as well as assay controls. We also looked at operational factors that would enable the assay to be easily implemented in our laboratory using widely available technology and readily transferable across laboratories. Lastly, determining the positive or negative status of a sample by using a generic cut point/threshold was essential for a plug-and-play assay so no experimental work would be necessary to establish a cut point for any new molecule.

The assays described by Stubenrauch et al. [13, 17] and Bautista et al. [14, 19] used in-house produced reagents. The assay that Stubenrauch et al. developed could measure total ADAs by adding the biotherapeutic to the sample to ensure immune complex formation. A biotinylated-murine anti-human IgG Fc was used to capture the complexes after binding to streptavidin-coated plates. Following appropriate washing steps, a monoclonal digoxigenylated anticynomolgus monkey IgG and polyclonal anti-digoxigenin-HRP were used for detection. As an assay control, a fusion of human IgG and cyno IgG was used. Bautista et al. [14, 19] developed the UNISA (Universal Indirect Species-Specific Immunoassay), an electrochemiluminescence assay where the ADAs in the sample were captured by the biotherapeutic coated on MSD (Meso Scale Discovery) plates. A commercial antispecies IgG Fc ruthenium conjugate was used as the detection reagent. A mouse anti-human IgG/cynomolgus monkey IgG1 chimeric monoclonal antibody was used as a positive control for monkey studies and similar chimeras were produced for studies in rat.

The assay that Stubenrauch et al. developed presented most of the qualities for the assay format that we were looking for. Moreover, the number of reagents, labeling of reagents, and assay steps could be simplified. Although the UNISA is a simple ELISA, it required the production of a chimeric mouse-cynomolgus monkey IgG assay control. Our laboratory has a key reagent, a mouse monoclonal antibody that is reactive to many Genentech humanized mAb biotherapeutics [20] that could be used to produce similar assay control chimeras. However, a broader binding specificity was desirable as this antibody showed limited binding to human derived antibodies. Moreover, producing a new clone with broader specificity was not an option at the time.

Our generic assay format is represented in Figure 1(b). It uses murine IgG1 monoclonal antibody, R10Z8E9, as the capture reagent (same as Stubenrauch et al.) directly coated on polystyrene 96-well microtiter plates. Thus, there was no need for streptavidin-coated plates and biotin conjugation of the antibody. R10Z8E9 specificity is directed against a conformation epitope on the CH2 domain of all four subclasses of human Fc gamma [17] and is available at Genentech. Regarding the detection antibody, Stubenrauch et al. produced a specific murine anticynomolgus monkey IgG mAb that was labeled with digoxigenin. A secondary mouse anti-DIG-HRP was necessary for detection. In contrast, our assay uses only one detection reagent that is commercially available conjugated to HRP. For assay positive control, a human IgG-cyno IgG conjugate was produced and used for routine sample analysis similar as described previously [13]. Again, human IgG and cynomolgus monkey IgG are readily available materials that can be easily conjugated. Furthermore, the ADC or the biotherapeutic of interest is another reagent for this assay that is available prior to starting the animal study and does not need to be conjugated unlike our default biotherapeutic-specific biotin-DIG ELISA.

Similar to the assays that Stubenrauch et al. and Bautista et al. developed, our assay is versatile and could be applicable to species other than cynomolgus monkeys by preparing the appropriate human IgG-species IgG fusion assay control and acquiring the antispecies detection reagent.

### 3.2. The Same Concentration of Biotherapeutic Can Be Used across Biotherapeutics to Form Complexes with ADA

The generic assay format detects complexes formed by ADAs with the biotherapeutic, which can already be present in circulation and could be detected in the assay. However, for our purposes the assay should detect not only ADAs already complexed with the biotherapeutic but also not-complexed ADAs. In the absence of biotherapeutic, the ADAs in the sample would not be detected. Therefore to ensure measurement of total ADAs in the sample, the biotherapeutic has to be added to the sample for the complex formation prior to the analysis. Hence, once the reagents and the assay format were established, experiments were conducted to determine the appropriate amount of biotherapeutic that was needed to add to the test samples in order to detect the total ADAs.

As this is a generic assay format with potential application not only to ADCs but also to other antibody-derived biotherapeutics, several types of molecules were included in the evaluation. Cynomolgus monkey pooled serum samples were prepared by adding 0, 0.5, 5, and 50 *μ*g/mL of ADA (purified cynomolgus monkey anti-human IgG) with 8 biotherapeutics at concentrations ranging from 0 to 20 *μ*g/mL. With all the biotherapeutics, an increase in signal was observed with increasing levels of ADAs and increasing concentrations of biotherapeutics. As more complexes with the ADAs were formed, a plateau was reached where no significant changes in signal were observed with varying concentrations of both ADAs and biotherapeutics. In all the cases, the highest signal observed was below the maximum reading absorbance limit of the microplate reader.

The amount of biotherapeutic to add to the samples was selected based on the ADA signal-to-background ratios observed for different biotherapeutics at various ADA levels. At each level of biotherapeutic, the ratios were calculated by comparing the signal in the presence of ADA with biotherapeutic to the signal with the same biotherapeutic amount in the absence of ADA.

**Figure 2:**
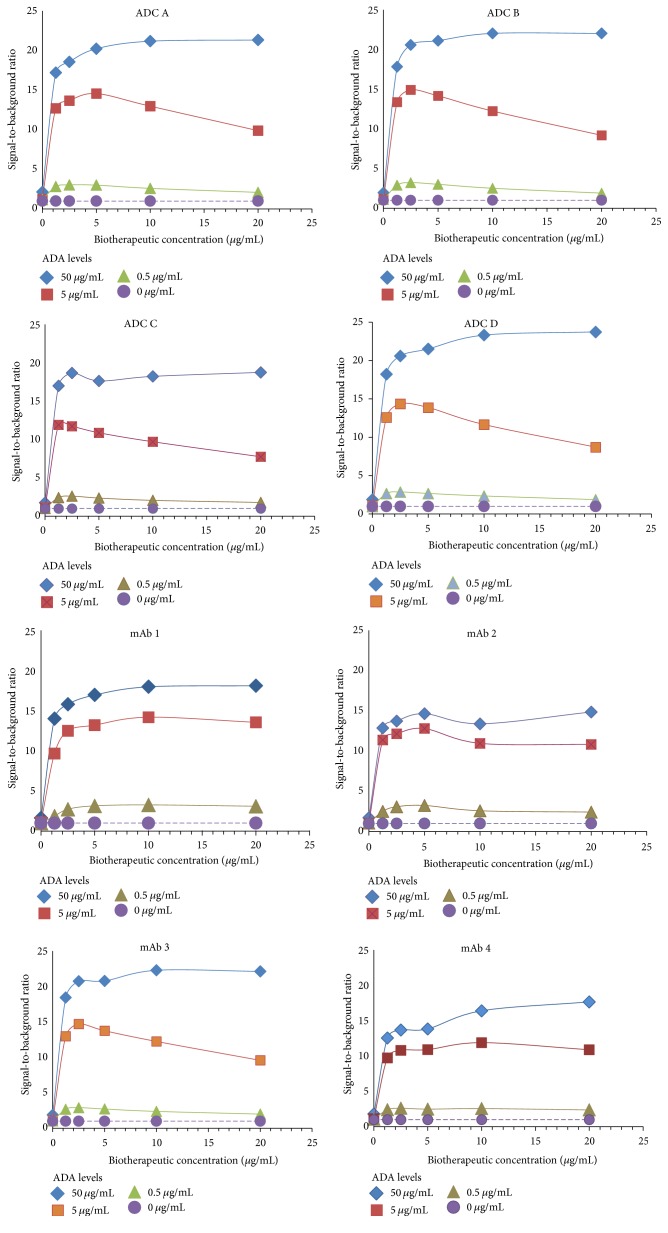
The same concentration of biotherapeutic can be used with different biotherapeutics to form complexes with ADA. Signal-to-background ratios obtained in a pooled cynomolgus monkey serum spiked with purified cynomolgus monkey anti-human IgG ADAs at 0 to 50 *μ*g/mL and with 8 biotherapeutics at 0 to 20 *μ*g/mL. The biotherapeutics consisted of 4 ADCs, 3 mAbs, and a one-arm antibody. With all the biotherapeutics, the signal-to-background ratios increased with increasing concentrations of ADA to reach a plateau at biotherapeutic concentrations between 2 and 10 *μ*g/mL. Based on these data, a biotherapeutic concentration of 5 *μ*g/mL in neat serum was determined to be appropriate to form complexes with ADA in the samples containing up to 50 *μ*g/mL of ADA, the highest level tested in our study. ADAs = anti-drug antibodies; ADC = antibody-drug conjugate; mAb = monoclonal antibody.

As Figure 2 shows, in general, the signal-to-background ratios at all ADA levels with the 4 ADCs and 4 mAb-derived biotherapeutics reached the highest ratio at concentrations between 2 and 10 *μ*g/mL with no significant changes in the ratios at higher concentrations. Based on these data, a generic biotherapeutic concentration of 5 *μ*g/mL in neat serum was determined to be the appropriate concentration to form complexes with ADA in the samples containing up to 50 *μ*g/mL of ADA, the highest level tested in our study.

**Figure 3:**
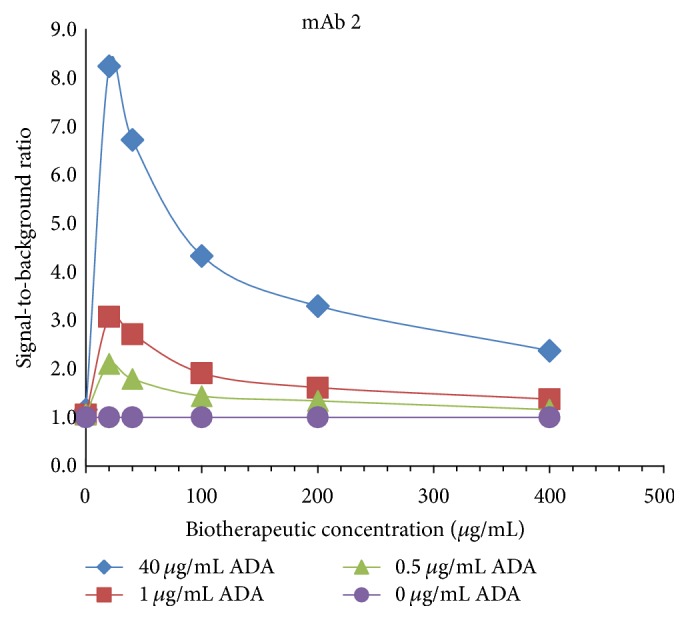
Early development experiments informed on biotherapeutic concentrations to test with a variety of molecules. Signal-to-background ratios obtained in a pooled cynomolgus monkey serum spiked with purified cynomolgus monkey anti-human IgG ADAs at 0, 0.5, 1, and 40 *μ*g/mL and with mAb 2 at 0, 20, 40, 100, 200, and 400 *μ*g/mL. The figure shows signal-to-background ratios increase with increasing concentrations of ADA. The highest signal-to-noise was observed at mAb 2 concentration of 20 *μ*g/mL while higher levels of mAb 2 yielded decrease in the signal-to-noise values at all the ADA levels tested. Based on these data, further experiments with a variety of molecules were performed with biotherapeutic levels up to 20 *μ*g/mL. ADAs = anti-drug antibodies; ADC = antibody-drug conjugate; mAb = monoclonal antibody.

Concentrations of biotherapeutics up to 400 *μ*g/mL were tested in early developmental experiments. As Figure 3 depicts, the signal-to-background ratios in samples spiked with various levels of ADA decreased at levels of the biotherapeutic higher than 20 *μ*g/mL.

**Figure 4:**
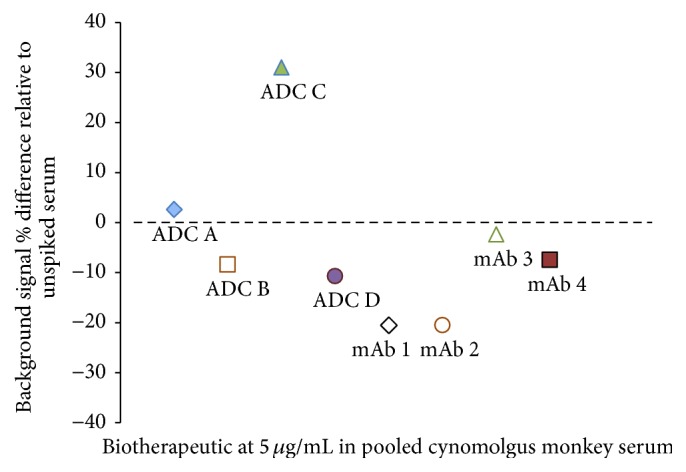
Background signal in pooled cynomolgus monkey serum differs across biotherapeutics in the generic assay. Eight biotherapeutics were added at 5 *μ*g/mL to a pooled cynomolgus monkey serum and tested in the generic assay. The background signal in the presence of biotherapeutic was compared to the signal in the unspiked sample and the percent difference calculated. The background signals differed across biotherapeutics with % differences compared to the unspiked pooled monkey serum ranging between −21% and 31%. ADC = antibody-drug conjugate; mAb = monoclonal antibody.

Figure 4 shows that, in absence of ADA, the background of the pooled cynomolgus monkey serum spiked with 5 *μ*g/mL of the 8 biotherapeutics above was between −21% and 31% of the unspiked serum. These differences in sample background with addition of biotherapeutic gave us a hint to expect similar differences across biotherapeutics when spiking samples from individual monkeys to evaluate biological variability in the assay. Moreover, differences in background signals across biotherapeutics would be a challenge to use the fusion positive control spiked into serum pool as a tool to determine sample positivity.

### 3.3. Background in Serum from Nontreated Cynomolgus Monkeys Differs with Each Biotherapeutic

Differences in background signal across molecules were observed not only in the pooled monkey serum but also in earlier assay development experiments in serum from individuals. Following industry general practices, screening cut point determination in our laboratory is determined based on the population background biological variability and normalized using a negative serum control [12]. We explored the feasibility of adopting a floating population generic cut point with an earlier version of the assay using commercial samples from 30 cynomolgus monkeys. For this approach to work, the population background should not change in the presence of the added biotherapeutic. However, different backgrounds were observed in unspiked samples and samples spiked with three mAbs. As a result, biotherapeutic-specific cut points were obtained for each mAb as summarized in Table 1. Using the cut point as determined from the population unspiked samples could result in high false positives with mAb 2 and mAb 5 but false negatives with mAb 6. Determining a biotherapeutic-specific cut point for each molecule was not a desirable characteristic for a plug-and-play assay. Thus, a different approach for estimating the cut point had to be followed.

### 3.4. Statistical Analysis and Determination of Individual Cut Point

Given the background and screening cut point differences across biotherapeutics, a generic approach based on a floating individual cut point [12] was explored. This involved using the baseline signal from each individual animal to establish its own cut point signal.

In this experiment, two samples taken 1 week apart from 15 cynomolgus monkeys were spiked at a concentration of 5 *μ*g/mL with 4 biotherapeutics: ADC C, mAb 1, mAb 2, and mAb 4. The background signal from each sample was determined in duplicate in the assay. A universal CPF was established based on the within monkey variability.

**Figure 5:**
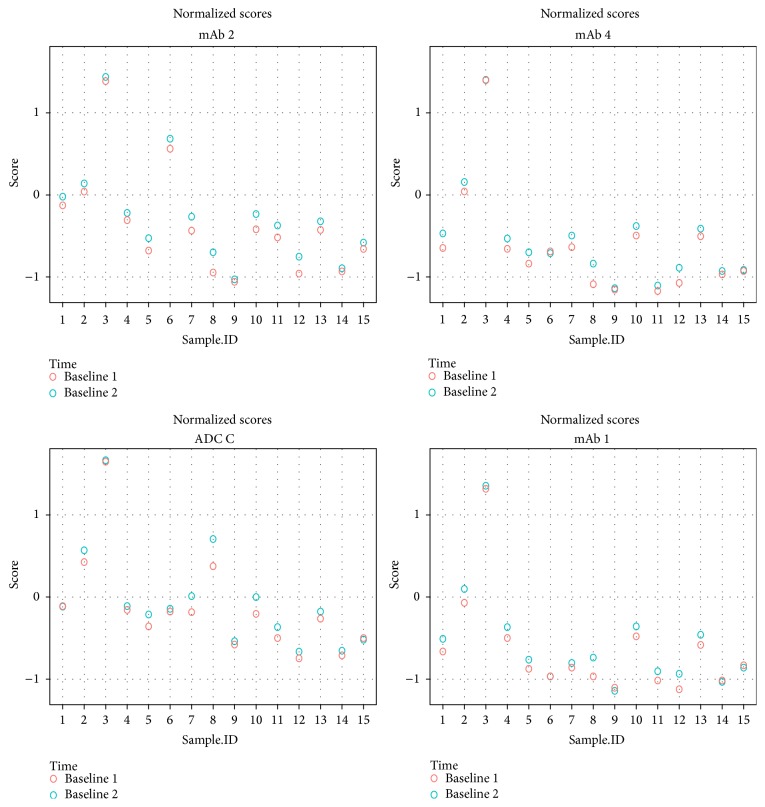
Normalized scores for background signal in samples from 15 cynomolgus monkeys across four biotherapeutics. Two samples taken 1 week apart from 15 cynomolgus monkeys were spiked at 5 *μ*g/mL with ADC C, mAb 1, mAb 2, and mAb 4 and analyzed in the generic assay. This figure illustrates the difference in responses for each biotherapeutic spiked in the individual samples. Normalized scores were calculated for each sample as the log (sample signal/negative control signal) across all four molecules.

Figure 5 shows the normalized scores calculated as the log (sample signal/negative control signal) across all four biotherapeutics in each of the two samples obtained from 15 cynomolgus monkeys. This figure illustrates the difference in responses for each biotherapeutic spiked in the individual samples. Although background signals differed across animals and molecules, within monkey variability was independent of the molecule.

Determination of individual signal cut points depends primarily on accurate assessment of (1) within monkey variability and (2) individual mean sample OD signal.  Table 2 summarizes the evaluation of the 4 biotherapeutics with differing structures but similar within monkey variability. The individual cut point signal method with any new mAb-derived biotherapeutic is based on the assumption that the generic cynomolgus monkey ADA assay will exhibit similar levels of within monkey variability with the new molecule.

Estimates of individual signal cut points (CP_*i*_) assume that we know the true underlying mean biotherapeutic naive sample absorbance signal. This is currently estimated using one or several observed sample absorbances prior to treatment by ${\bar{x}}_{i}$. The variability in this estimate is SD/*n* ^0.5^ where *n* is the number of observed sample absorbances used to estimate the mean. It is recommended to use several observations in estimating ${\bar{x}}_{i}$ in order to minimize this variability. Failure to do so can result in more false positives than expected from a given level of *α*.  Table 3 summarizes the estimated CPF based on the false positive rate *α* (1%, 5%, and 10%). In the experiments presented here we have used a cut point factor targeting a false positive rate of 5% for consistency with our previous studies. However, lower positive rates are adequate based on the primary purpose of immunogenicity testing in nonclinical studies [21].

With this cut point generic approach, induced and enhanced responses are not differentiated because by definition all the baseline signals are considered negative. Additionally, for each animal the postbaseline samples have to be analyzed with the baseline samples to determine the cut point signal.

Although floating individual cut points are not commonly used for immunogenicity assays, this approach could be also valuable for molecule specific nonclinical and clinical assays. For example, screening assays with high baseline signal differences across individuals could result in high cut point factors when applying the widely used floating population cut point. As a consequence, the assay might not have acceptable sensitivity and drug tolerance. Using the floating individual cut point approach has the requirement of an additional sample collection, as two biotherapeutic naive samples are needed from each subject (ideally for both, assay validation and study samples) to assess individual signal variability.

### 3.5. A Generic Assay Format Can Detect ADAs against the Cytotoxic Drug Domain of an ADC

Immune responses to an ADC include ADAs against its different domains, such as the monoclonal antibody and the linker-cytotoxic drug. The generic assay format uses the biotherapeutic as the reagent to form complexes and it is expected that ADAs against different ADC domains will be detected. A proof of concept model assay was used to demonstrate this point. Since the affinity purified positive control used for the assay characterization binds only to the mAb domain of an ADC, it could not be used to demonstrate that ADAs against the linker-drug can be detected. A cynomolgus monkey derived anticytotoxic drug antibody was not available in our laboratory, but we had a mouse monoclonal anticytotoxic drug mAb. An assay model was needed as the mouse ADA control required changing the detection and capture reagents in the generic assay format. As a consequence, a sheep anti-human IgG was used as the capture reagent and an HRP-conjugated goat anti-mouse IgG was used for detection.

**Figure 6:**
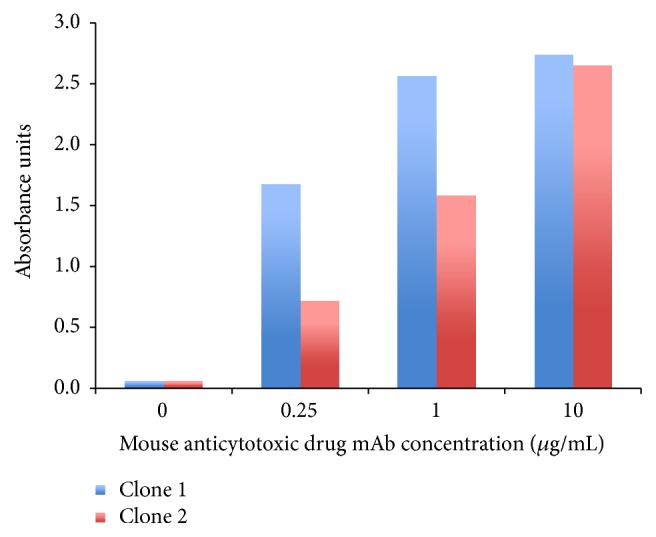
The generic assay detects ADAs against the linker-cytotoxic drug domain of an ADC. Two mouse anticytotoxic drug mAbs at various concentrations were spiked in pooled cynomolgus monkey serum with ADC C (5 *μ*g/mL). The reactivity of the immune complexes was measured in a generic assay model: the ADA-ADC complexes were captured on a sheep anti-human IgG-coated plate and for detection an anti-mouse IgG-HRP antibody was used. Positive responses were obtained with the two mAb clones at all the concentrations tested. ADA = anti-drug antibody; mAb = monoclonal antibody.

In this model assay, two mouse anticytotoxic drug mAbs (test ADA) at concentrations of 0.25, 1, and 10 *μ*g/mL were spiked in pooled cynomolgus monkey serum with ADC C at 5 *μ*g/mL. The ADA-ADC complexes were captured on a sheep anti-human IgG-coated plate. To detect the complexes, an anti-mouse IgG-HRP antibody was used.  Figure 6 shows the positive response obtained with the two test ADA murine antibodies. These data demonstrate that ADAs against the cytotoxic drug domain of an ADC can be detected in this generic assay format.

### 3.6. Evaluation of Relative Sensitivity, Drug Tolerance, and Reproducibility

Immunogenicity assays should have sufficient sensitivity to detect ADAs in the presence of the biotherapeutic in circulation. It is well understood that assay sensitivity is relative to the ADA surrogate control used in the assay. It may not represent the true sensitivity of the assay in test samples. For nonclinical species, a relative sensitivity of 1000 ng/mL or better is recommended [11, 12]. Although there are no numeric recommendations regarding drug tolerance, our goal was for the assay to be able to detect ADAs in the presence of expected levels of biotherapeutic in the sample predicted from the PK.

**Figure 7:**
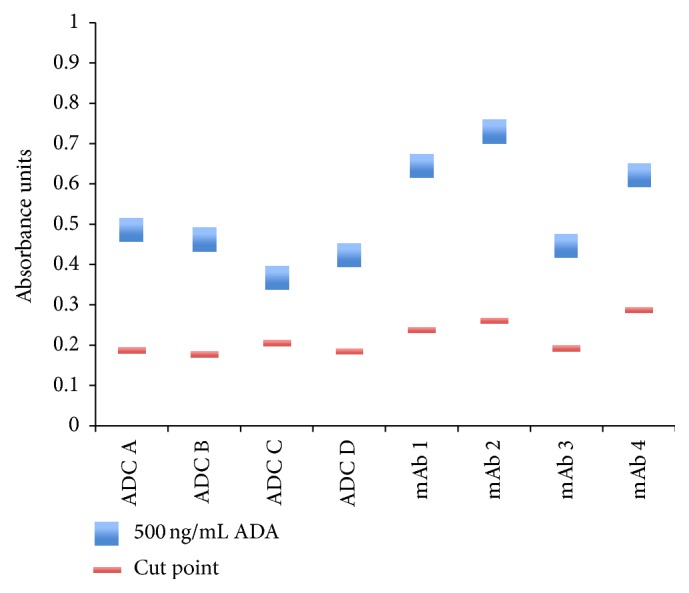
The plug-and-play assay showed adequate sensitivity for cynomolgus monkey studies across molecules. Pooled cynomolgus monkey serum was spiked with 5 *μ*g/mL of eight biotherapeutics (baseline sample) or with the biotherapeutics (5 *μ*g/mL) and 500 ng/mL of ADAs (purified cynomolgus monkey anti-human IgG). Cut points were calculated for each biotherapeutic from the signal at baseline and the generic cut point factor for a 5% false positive rate (CPF = 1.16). With all the biotherapeutics, the sample containing 500 ng/mL of ADA was positive with signals above their corresponding cut points. ADA = anti-drug antibody; ADC = antibody-drug conjugate; mAb = monoclonal antibody.

The plug-and-play assay showed excellent relative sensitivity and drug tolerance suitable for the needs of the studies. Using the human IgG-cyno IgG fusion positive control, the assay sensitivity was 41 ng/mL. It is expected that sensitivity values with different biotherapeutics and ADA sources may vary. Sensitivity experiments were not performed with different biotherapeutics. However, the data from the experiment illustrated in Figure 2 was used to demonstrate that the assay sensitivity across biotherapeutics was better than 500 ng/mL of ADA (purified cynomolgus monkey anti-human IgG). A cut point signal was calculated for each biotherapeutic from the background signal with 5 *μ*g/mL of biotherapeutic (baseline sample) and the CPF for a 5% false positive rate (1.16).  Figure 7 shows that, with all eight biotherapeutics, the sample containing 500 ng/mL of ADA was positive with signals above their corresponding cut points. These data indicated that the plug-and-play assay had adequate sensitivity across molecules to detect ADAs in cynomolgus monkey studies.

The drug tolerance was determined using purified cynomolgus monkey anti-human IgG positive control source spiked at 1 *μ*g/mL in cynomolgus monkey pooled serum and varying concentration of one of the biotherapeutics (mAb 2) from 4 to 500 *μ*g/mL. The assay was capable of detecting 1 *μ*g/mL of the ADA positive control in the presence of up to 440 *μ*g/mL of mAb 2.

The assay precision was evaluated by the interassay run for the signal of the negative control (unspiked pooled cynomolgus monkey serum) and the relative ADA values generated for the positive control (fusion human IgG-cyno IgG molecule).  Table 4 shows that the assay is reproducible with the coefficient of variation values for the negative and positive controls below 16%.

### 3.7. Analysis of Samples from Studies in Cynomolgus Monkey and Comparison to Data from the Biotherapeutic-Specific ELISA

Samples from two studies in cynomolgus monkeys with ADCs A and B and their respective mAbs (A and B) were analyzed in the plug-and-play assay. The results were compared to the data obtained initially in the biotherapeutic-specific biotin-DIG ELISA. A 5% false positive rate CPF was used for the plug-and-play assay to resemble the approach followed to calculate the population cut point factor in the biotin-DIG ELISA. Immunogenicity incidence and the relative ADA levels in samples that screened positive were compared.

**Figure 8:**
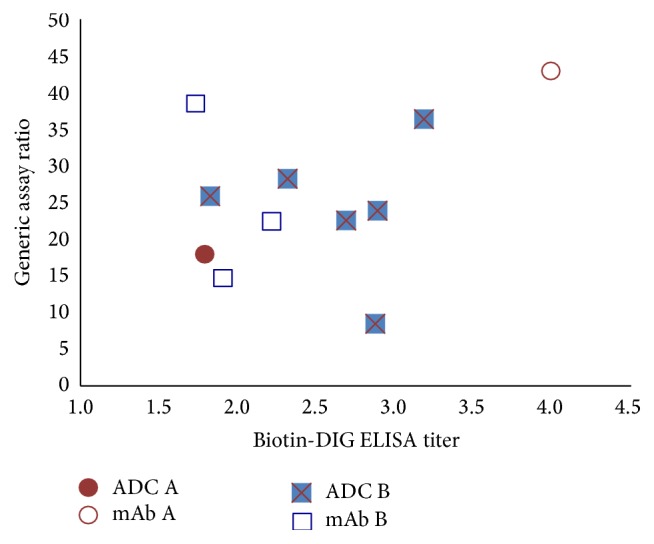
Good agreement between ADA titers by biotin-DIG ELISA and relative ADA ratios by the plug-and-play assay. The figure summarizes the relative levels of ADA Studies 1 (ADC A and mAb A) and 2 (ADC B and mAb B) whose samples were determined as positive by two ADA methods. The ratios calculated in the plug-and-play assay were plotted versus the titers determined in the biotherapeutic-specific biotin-DIG ELISAs. Overall, good agreement between the two procedures was observed.

Table 5 summarizes the immunogenicity results obtained in the two studies with the two assays. Samples from Study 1 with both ADC A and mAb A were all negative at baseline in the biotin-DIG ELISA. In the plug-and-play assay, by definition, samples are always negative at baseline. At postbaseline time points, the plug-and-play assay detected ADAs in the two animals that had ADA positive results with the biotin-DIG ELISA. Moreover, the relative levels calculated by the ratio agreed with the titers determined in the specific biotin-DIG ELISA as Figure 8 illustrates. However, the plug-and-play assay detected more positive responses than the biotherapeutic-specific biotin-DIG ELISA. With ADC A, two more animals had postbaseline positive signals in the plug-and-play assay than in the biotin-DIG ELISA, although the signals were not very strong (Table 6). With mAb A, the plug-and-play assay detected positive responses at days 15 and 44 (ratios of 2.5 and 12.3, resp.) in one animal that was negative in the biotin-DIG ELISA. In addition, the plug-and-play assay detected ADAs earlier (at day 15) and with a stronger signal (at a ratio of 38) in the only animal dosed with mAb A that was also positive in the biotin-DIG ELISA.

In Study 2 with both ADC B and mAb B, all the baseline samples were negative in the specific biotin-DIG ELISA and all the animals had positive responses at day 43 after baseline. Likewise, in the plug-and-play assay, all the animals produced postbaseline positive signals (Table 5). Figure 8 shows general good agreement between the ADA titers determined in the specific biotin-DIG ELISA and the relative levels calculated by the ratio in the plug-and-play assay. There were two samples that deviated from the others. One postbaseline sample from a monkey receiving mAb B had a low ADA titer in the Biotin-DIG ELISA but a high ADA ratio in the plug-and-play assay. Conversely, a monkey receiving ADC B produced a high ADA titer in the biotin-DIG ELISA and low ratio in the plug-and-play assay.

It is not uncommon to observe differences between immunogenicity data obtained from different ADA assays specially when using different formats, screening cut point, and statistical approaches for data analysis [22]. In our studies, the assay format, the approach to cut point determination, and the types of ADAs measured were all different. In the biotin-DIG ELISA, the ADAs need to be fully unbound from the biotherapeutic so they can actively react with the two biotherapeutic-conjugated reagents. Appropriate assay conditions (e.g., in-solution phase and long incubation time) favor binding of the ADAs in the sample to the conjugate reagents in the presence of the biotherapeutic in the sample. In the plug-and-play assay, only biotherapeutic-ADA complexes are detected with the ADAs bound to the biotherapeutic through one or two arms and the assay reagents recognizing the complexes. The biotin-DIG ELISA can detect ADAs with different isotypes while the plug-and-play assay can detect only ADAs of the IgG isotype. The biotin-DIG ELISA relies on biotherapeutic-conjugated reagents where the labels may potentially mask ADA epitopes and interfere with bridging of the reagents while the plug-and-play assay uses the unlabeled biotherapeutic as reagent. Antibody affinity can also play a role in the differences observed between assays. As a consequence, the assays may vary in their sensitivity to detect ADAs and drug tolerance in the study samples, explaining the differences observed in the Study 1 screening data between the two assays.

## 4. Conclusions

Assessment of immunogenicity in banked samples from early nonclinical studies may be triggered by PK and safety data at short notice. The availability of a plug-and-play assay without the need for assay optimization and molecule specific cut points is highly valuable during drug discovery where immunogenicity may not be assessed on a routine basis.

We developed a plug-and-play assay, where the assay format and data analysis are generic. This assay is suitable for ADCs as well as other antibody-derived biotherapeutics. In addition, screening of samples and, if positive, ADA relative level determination can be performed in the same run. Sample testing from two studies in cynomolgus monkey showed that the plug-and-play assay was able to detect equal or greater number of positive samples than the biotherapeutic-specific biotin-DIG ELISA. Therefore, from this pilot study one could assume that animals with a positive ADA response would not be missed by moving to the plug-and-play assay. This approach is valuable to streamline immunogenicity assessments for molecules in the discovery phase of drug development.

## Figures and Tables

**Table 1:** Difference in background responses across three biotherapeutics resulted in molecule specific screening cut points following a floating population data approach. Serum samples from 30 cynomolgus monkeys were spiked with 3 mAbs in early assay development experiments. The unspiked and mAb-spiked samples were analyzed in the generic assay format and a floating population screening cut point was evaluated. Different backgrounds were observed in the samples spiked with three mAbs compared to the unspiked corresponding samples. Therefore, molecule specific screening cut points were obtained for each mAb.

	Unspiked	mAb 2	mAb 5	mAb 6
Cut point (AU)	0.241	0.263	0.426	0.190

AU = absorbance units.

**Table 2:** Estimation of within monkey variability across biotherapeutics. Four types of biotherapeutics were spiked in two baseline samples taken 1 week apart from 15 cynomolgus monkeys and tested in the assay. The background data from the biotherapeutic spiked samples showed that within monkey signal variability was similar. Therefore, the overall variability obtained with these four molecules could be used to estimate a generic cut point factor.

Biotherapeutic	Type of molecule	Estimated within monkey SD
ADC C	ADC	0.095
mAb 1	One-arm mAb	0.086
mAb 2	IgG1 mAb	0.096
mAb 4	IgG4 mAb	0.087
Overall		0.091

SD = estimate of within monkey signal variability.

**Table 3:** Estimation of cut point factor based on different false positive rates (*α*). Cut point factor (CPF) calculated at different false positive rates based on the within monkey variability observed across 4 biotherapeutics. This generic CPF can be used to establish the floating individual cut point signal according to each cynomolgus monkey baseline signal.

*α*	Cut point factor
0.10	1.12
0.05	1.16
0.01	1.24

*α* = false positive rate.

**Table 4:** Reproducibility of the plug-and-play assay. Assay control precision expressed as the coefficient of variation of the signal observed in 6 independent runs. The negative control is a pooled cynomolgus monkey serum. The positive control is a human IgG-cynomolgus monkey IgG fusion molecule. Interassay precision for signal values obtained with the two controls is summarised below. In addition, a ratio of the signals obtained with the positive and negative controls was calculated for assay performance evaluation.

Assay control	Number of runs	Mean measurement (units)	CV (%)
Negative control	6	0.169 (AU)	16

Positive control	6	0.302 (AU)	13
		1.80 (ratio)	11

Fusion positive control at 125 ng/mL.

AU = absorbance units; CV = coefficient of variation.

Ratio = positive control signal/negative control signal.

**Table 5:** Summary of immunogenicity data for cynomolgus monkey Studies 1 and 2 with the biotin-DIG ELISA and the plug-and-play assay. Samples from cynomolgus monkey studies with two ADCs and their corresponding mAbs were analyzed in the plug-and-play assay. The number of animals that developed anti-drug antibodies after exposure to the above molecules and the immunogenicity incidence obtained with this assay were compared to those previously obtained in the biotherapeutic-specific biotin-DIG ELISA. In Study 1, the plug-and-play assay detected the same positive responses as the biotin-DIG ELISA plus three additional animals with ADA positive responses. In Study 2, all the monkeys had positive responses with the biotin-DIG ELISA and the plug-and-play assay.

Study	Molecule	Positive of total		Immunogenicity incidence	
		Biotin-DIG	Plug-and-play	Biotin-DIG	Plug-and-play
1	mAb A	1 of 4	2 of 4	25%	50%
1	ADC A	1 of 4	3 of 4	25%	75%
2	mAb B	3 of 3	3 of 3	100%	100%
2	ADC B	6 of 6	6 of 6	100%	100%

ADAs = anti-drug antibodies; ADC = antibody-drug conjugate; mAb = monoclonal antibody.

**Table 6:** Postbaseline ADA positive responses in cynomolgus monkey Study 1 as detected in the biotin-DIG ELISA and the plug-and-play assay. In Study 1, the biotin-DIG ELISA detected ADAs in two cynomolgus monkeys, one of them dosed with ADC A and the second one with mAb A. These two animals also had positive signals in the plug-and-play assay with good agreement between the ADA titer determined in the biotin-DIG ELISA and the relative ADA level by the ratios calculated in the plug-and-play assay (Figure 8). However, the plug-and-play assay detected ADA responses in animal 1 dosed with mAb A earlier (day 15) than the biotin-DIG ELISA. The plug-and-play assay identified positive responses in 3 additional monkeys, one dosed with mAb A and two dosed with ADC A.

Molecule	Animal	Time point	Biotin-DIG ELISA		Plug-and-play assay	
			Screening result (positive/negative)	AU or titer	Screening result (positive/negative)	AU or ratio
mAb A	1	Baseline	Negative	0.054 AU	Negative	0.067 AU
		D15	Negative	0.067 AU	Positive	Ratio of 37.9
		D44	Positive	Titer of 3.99	Positive	Ratio of 42.9

mAb A	2	Baseline	Negative	0.057 AU	Negative	0.196 AU
		D15	Negative	0.065 AU	Positive	Ratio of 2.5
		D44	Negative	0.061 AU	Positive	Ratio of 12.3

ADC A	3	Baseline	Negative	0.065 AU	Negative	0.117 AU
		D15	Negative	0.057 AU	Positive	Ratio of 1.5
		D44	Negative	0.052 AU	Positive	Ratio of 1.3

ADC A	4	Baseline	Negative	0.060 AU	Negative	0.149 AU
		D15	Negative	0.053 AU	Negative	0.119 AU
		D44	Positive	Titer of 1.79	Positive	Ratio of 18.0

ADC A	5	Baseline	Negative	0.065 AU	Negative	0.128 AU
		D15	Negative	0.052 AU	Negative	0.119 AU
		D44	Negative	0.061 AU	Positive	Ratio of 3.5

ADAs = anti-drug antibodies; ADC = antibody-drug conjugate; AU = absorbance units; D = days after dosing or after baseline; mAb = monoclonal antibody; R = ratio.

Ratio = (postbaseline AU/baseline AU).
